# Long-Term Abdominal Drains in Refractory Ascites Due to Cirrhosis: A Retrospective Cohort Analysis

**DOI:** 10.7759/cureus.97259

**Published:** 2025-11-19

**Authors:** Mohammad Armaghan Farooq Dar, Raaid Jamil, Saeed Alhakeem, Abdul Khan, Ilsa Awan, Lynne Kendrick, Muhammad Iftikhar

**Affiliations:** 1 Department of Gastroenterology, Royal Oldham Hospital, Oldham, GBR

**Keywords:** cirrhosis, hospital readmissions, length of stay, liver failure, long-term abdominal drain, palliative hepatology, quality improvement, refractory ascites, spontaneous bacterial peritonitis

## Abstract

Background: Refractory ascites in cirrhosis presents major management challenges and is associated with frequent hospital admissions, reduced quality of life, and poor survival. Long-term abdominal drains (LTADs) have emerged as a palliative alternative to repeated large-volume paracentesis (LVP), yet real-world outcome data remain limited.

Methods: We performed a retrospective analysis of 44 patients with cirrhosis and refractory ascites who underwent LTAD insertion between 2019 and 2024 at a district general hospital. Clinical outcomes, including survival, complication rates, hospital admissions, and length of stay (LOS), were evaluated. Data were analysed using Kaplan-Meier survival curves and descriptive statistics for pre- and post-LTAD comparisons.

Results: Median survival after LTAD was eight months; Kaplan-Meier estimated 12-month survival was 65%. LTAD insertion was associated with a 47% reduction in hospital admissions and a 55% reduction in cumulative LOS in the six months post-procedure compared to the six months pre-procedure. Specifically, admissions dropped from 102 to 54, and LOS from 1,150 to 520 days for the cohort. Spontaneous bacterial peritonitis (SBP) occurred in 18% of patients and catheter-related infections in 11%. Ascitic fluid leakage was observed in 20% and drain blockage in 14%. No procedure-related mortality was identified.

Conclusion: LTADs represent a safe and effective palliative strategy for managing refractory ascites in cirrhosis. They significantly reduce healthcare utilisation while maintaining acceptable complication rates. Careful patient selection and multidisciplinary management are essential to optimise outcomes. LTADs are currently a case-by-case palliative option, but ongoing trials will further clarify their role. Prospective studies are warranted to validate these findings and to assess quality-of-life improvements.

## Introduction

Refractory ascites is a debilitating complication of decompensated cirrhosis, occurring in approximately 10% of patients, and is associated with reduced survival and impaired quality of life [1,2]. Standard management strategies include repeated large-volume paracentesis (LVP) with albumin infusion or consideration of transjugular intrahepatic portosystemic shunt (TIPS) [3]. However, both approaches have limitations: LVP requires frequent hospital visits and may contribute to protein depletion, while TIPS carries risks of hepatic encephalopathy and is often contraindicated in frail patients [4]. Another palliative approach involves automated low-flow peritoneal shunt devices (alfapump), which can reduce the need for paracentesis but have shown significant complication rates and cost issues [5].

Long-term abdominal drains (LTADs) have emerged as a palliative option, particularly for patients unsuitable for TIPS or transplantation [6]. Early case series and feasibility studies suggested that LTADs can effectively control ascites while reducing the burden of repeated hospital-based paracentesis [7,8]. More recent studies and observational cohorts indicate that LTADs may reduce healthcare utilization alongside costs and allow community-based ascites management [9,10]. A recent systematic review confirmed reductions in hospital admissions and improved patient autonomy, although concerns regarding infection remain [7]. Despite growing clinical interest, evidence for LTAD use in cirrhosis remains limited compared to their established role in malignant ascites [11]. Current guidelines and expert consensus do not consider LTADs a standard of care in cirrhosis, advising use on a case-by-case basis pending further evidence [12].

This study presents a single-centre retrospective analysis of outcomes following LTAD insertion for refractory ascites due to cirrhosis, focusing on survival, infection rates, and hospital utilization. Refractory ascites remains a major cause of hospitalization in advanced cirrhosis and confers a poor prognosis [13]. By examining real-world outcomes with LTADs, we aim to inform their role as a palliative intervention in this challenging patient population. The primary objective of this study was to assess clinical outcomes in patients with refractory ascites due to cirrhosis who underwent LTAD insertion. We evaluated survival, complications, and healthcare utilisation-specifically hospital admissions and length of stay-before and after LTAD placement. This analysis aimed to provide service-level insights into the safety and utility of LTADs as a palliative intervention in advanced cirrhosis.

## Materials and methods

Study design and patients

This retrospective cohort included adult patients with cirrhosis and refractory ascites who underwent LTAD insertion between January 2019 and March 2024 at Royal Oldham Hospital, a District General Hospital in the UK. Data were extracted from the hospital’s electronic health record (EHR) system and verified against procedural notes. Refractory ascites was defined as ascites unresponsive to dietary sodium restriction and diuretic therapy, or ascites that is diuretic-intolerant, with patients requiring three or more therapeutic paracenteses within a three-month period (meeting criteria for diuretic intractability). Patients with hepatocellular carcinoma (HCC) beyond Milan criteria, ongoing systemic infection/sepsis at the time of consideration, or other contraindications to LTAD placement (e.g., multiloculated ascites, severe uncorrectable coagulopathy, or abdominal adhesions) were excluded. All patients were under the care of a hepatology multidisciplinary team (MDT); LTADs were considered only for those ineligible for TIPS or liver transplantation due to clinical factors (active alcohol use, comorbidities, advanced age, etc.). The sample size (n=44) reflected all consecutive patients meeting inclusion criteria during the study period, ensuring complete case capture rather than pre-determined power calculation. Missing data were minimal (<5%) and handled by case-wise exclusion for affected variables.

LTAD procedure

LTAD insertions were performed under ultrasound guidance by interventional radiologists or experienced hepatologists at the bedside. A tunneled peritoneal catheter (10-French) with a one-way valve mechanism was used in all cases (Rocket® Medical LTAD system, Washington, UK). The catheter was placed in the right or left lower quadrant under local anaesthesia, with prophylactic intravenous antibiotics given per hospital protocol prior to insertion. Patients and caregivers received education on drain care and aseptic technique. Community nursing services were engaged for regular home visits to assist with drainage as needed. After insertion, patients were typically allowed to drain ascitic fluid intermittently at home (e.g., 1-2 L three times per week) based on symptom relief, with careful monitoring to avoid excessive fluid removal in a short time frame. All patients were maintained on diuretics as tolerated, and norfloxacin was prescribed for spontaneous bacterial peritonitis (SBP) prophylaxis in those with low ascitic protein or prior SBP, per standard practice.

Data collection

Baseline demographic, clinical, and laboratory data were collected from electronic health records (EHR), including age, sex, aetiology of cirrhosis, Child-Pugh and Model for End-Stage Liver Disease (MELD) scores, and prior complications such as SBP or hepatorenal syndrome (HRS). Outcomes assessed included overall survival after LTAD insertion, frequency of all-cause hospital admissions, cumulative hospital length of stay (LOS), and procedure-related or infectious complications. We specifically tracked instances of SBP (defined by ascitic fluid neutrophils > 250/mm³ and positive cultures or treated as such), catheter tract infections (cellulitis or tunnel infection requiring antibiotics), significant ascitic fluid leakage around the catheter site, and catheter blockage or dysfunction. We also noted if any patients underwent liver transplantation or TIPS after LTAD placement.

Statistical analysis

Survival analysis was performed using the Kaplan-Meier method, with follow-up defined from date of LTAD insertion to death, transplant, or last follow-up (censoring date: June 30, 2024). Median survival and 12-month survival probability with 95% confidence intervals (CI) were calculated. Hospital utilization in the six months before versus after LTAD insertion was compared descriptively. We totalled all inpatient admissions and hospital days for each patient in the specified time frames. Given the before-and-after design and the possibility that some patients died before reaching six months post-LTAD, results were primarily reported as aggregate changes. A paired statistical test (Wilcoxon signed-rank test) was conducted on the number of admissions per patient before vs. after LTAD as a supportive analysis; a two-tailed p < 0.05 was considered statistically significant. Categorical variables (e.g., complication rates) were reported as proportions. All analyses were conducted using IBM SPSS Statistics version 27 (IBM Corp., Armonk, NY, USA). Missing data were handled by case-wise exclusion. Given the descriptive and service-evaluation nature of the study, formal sample size estimation and inferential modelling were not undertaken.

Ethical considerations

The study was conducted under the institutional clinical governance framework, which confirmed that it met the criteria for a service evaluation and waived the requirement for individual patient consent. All data were anonymized prior to analysis, and all procedures adhered to the principles of the Declaration of Helsinki and local NHS governance policies.

## Results

Patient characteristics

A total of 44 patients underwent LTAD insertion during the study period. The median age was 63 years (interquartile range [IQR] 57-71), and 61% were male. The most common aetiology of cirrhosis was alcohol-related liver disease (24 patients, 55%), followed by non-alcoholic steatohepatitis (NASH) in nine (20%), viral hepatitis in five (11%), and other causes (autoimmune, cryptogenic, cardiac, or mixed aetiologies) in six (14%). At baseline, 80% of patients were Child-Pugh class C, and 20% were class B, with a median MELD-Na score of 18 (IQR 15-22). All patients had undergone at least one LVP prior to LTAD insertion (median of 4 procedures in the preceding three months). Five patients (11%) had been evaluated for TIPS but were deemed unsuitable due to either refractory hepatic encephalopathy (n=3) or cardiac contraindications (n=2). None were active candidates for liver transplantation at the time of LTAD insertion, primarily due to comorbidities or ongoing alcohol use.

Table 1 summarises the baseline characteristics of the study cohort.

**Table 1:** Baseline Characteristics of Patients Undergoing Long-Term Abdominal Drain (LTAD) Insertion Values are presented as number (percentage) or median (interquartile ranges, IQR) unless otherwise specified. TIPS: transjugular intrahepatic portosystemic shunt; MELD-Na: Model for End-Stage Liver Disease incorporating sodium.

Variable	Value / Frequency
Total patients, n	44
Median age (IQR)	63 years (57–71)
Male, n (%)	27 (61%)
Aetiology of cirrhosis :	
Alcohol-related liver disease	24 (55%)
Non-alcoholic steatohepatitis (NASH)	9 (20%)
Viral hepatitis	5 (11%)
Other causes	6 (14%)
Child-Pugh class:	
Class B	9 (20%)
Class C	35 (80%)
Median MELD-Na score (IQR)	18 (15–22)
Median number of paracenteses (past 3 months)	4
Evaluated for TIPS, n (%)	5 (11%) — all deemed unsuitable

Survival outcomes

The median survival following LTAD insertion was eight months (95% CI 5-11). The Kaplan-Meier estimated 12-month survival was 65% (95% CI 50-80). At study censoring (June 2024), 27 of 44 patients (61%) had died with the LTAD in situ, while 17 patients (39%) were alive at follow-up. Importantly, no deaths were directly attributable to the LTAD procedure.

**Figure 1:**
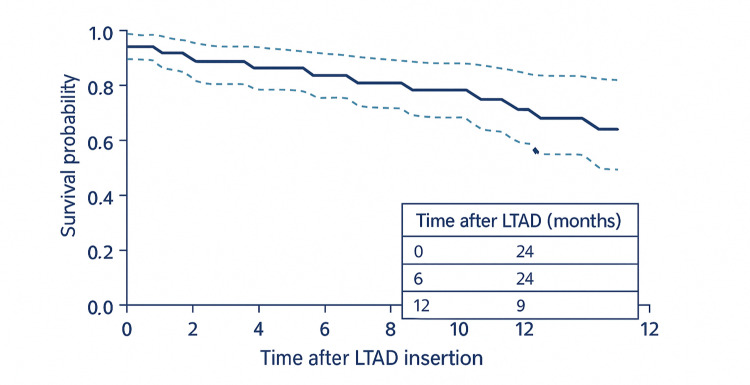
Kaplan–Meier Survival Curve Following Long-Term Abdominal Drain (LTAD) Insertion Kaplan–Meier survival analysis for 44 patients with refractory ascites secondary to cirrhosis following LTAD insertion. The median survival was eight months, and the estimated 12-month survival probability was 65%. Dashed lines represent 95% confidence interval bounds. Two patients alive beyond 12 months were censored at last follow-up.

Table 2 outlines the survival outcomes, and Figure 1 presents the Kaplan-Meier survival curve.

**Table 2:** Survival Outcomes Following Long-Term Abdominal Drain (LTAD) Insertion CI: confidence interval. Survival was calculated using the Kaplan–Meier method. Follow-up defined from LTAD insertion to death, or last review.

Outcome	Result
Median survival, months (95% CI)	8 (5–11)
12-month survival probability, %	65% (95% CI 50–80%)
Deaths directly attributable to LTAD	0 (0%)
Median follow-up time, months	10 (range 1–18)

Complications

LTAD-related complications are summarised in Table 3. SBP occurred in eight patients (18%), while catheter tract infections (cellulitis at the exit site) occurred in five (11%). All infections were managed successfully with antibiotics; no cases of fungal peritonitis or peritoneal abscess were identified. Mechanical issues included ascitic fluid leakage in nine patients (20%), generally managed conservatively with reinforced dressings, and drain blockage in six (14%), which were treated with catheter flushes or elective replacement after several months. No cases of bowel perforation, haemorrhage, or procedure-related mortality were observed. One patient (2%) had the LTAD removed after persistent leakage and resumed intermittent LVP.

**Table 3:** Complications Following Long-Term Abdominal Drain (LTAD) Insertion Complications were classified as infectious (spontaneous bacterial peritonitis, catheter-related infection) or mechanical (leakage, blockage). No cases of bowel perforation or procedure-related mortality were reported.

Complication	Number of Patients (%)
Spontaneous bacterial peritonitis	8 (18%)
Catheter-related infection (cellulitis)	5 (11%)
Ascitic fluid leakage (persistent)	9 (20%)
Drain blockage (requiring intervention)	6 (14%)
Bowel perforation	0 (0%)
Procedure-related death	0 (0%)

Healthcare utilisation

The use of LTADs was associated with a marked reduction in healthcare utilisation in the six months following insertion compared with the six months prior. The total number of hospital admissions decreased from 102 to 54, representing a 47% reduction, while the cumulative hospital LOS fell from 1,150 days to 520 days - a 55% reduction. Fifteen patients (34%) required no hospital readmissions in the six months post-LTAD, reflecting effective community-based ascites management. The median number of admissions per patient decreased from two to one, a statistically significant change on paired analysis (p = 0.01). Median survival following LTAD insertion was eight months (95% CI 5-11), consistent with the Kaplan-Meier estimate. Even after excluding five patients who died within six months post-procedure, the reduction in admissions and LOS remained consistent (approximately 40% reduction). No readmission was related to procedure-related complications beyond those listed under infection outcomes.

**Figure 2:**
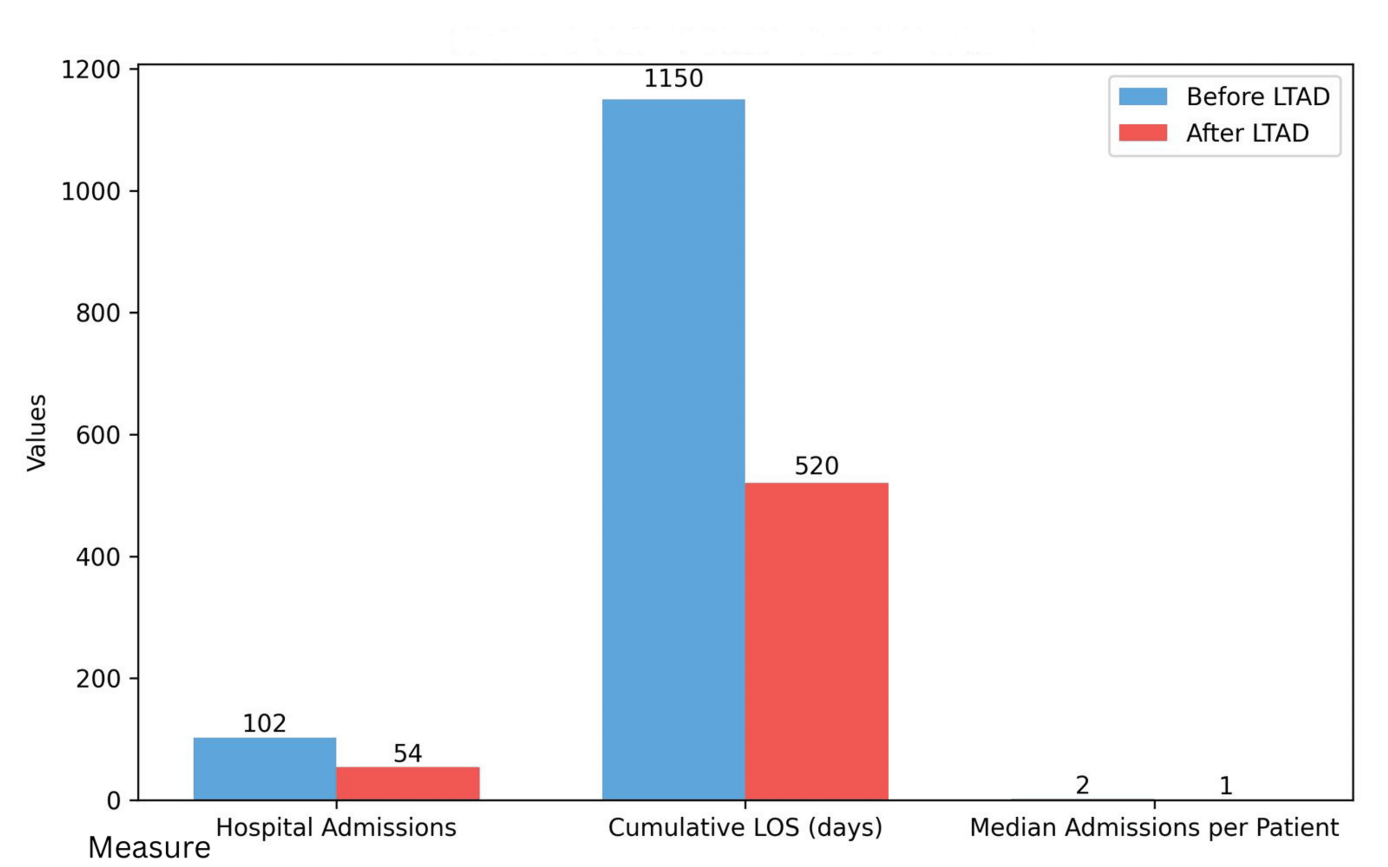
Change in Hospital Utilisation Six Months Before and After Long-Term Abdominal Drain (LTAD) Insertion The use of LTADs was associated with a reduction in total hospital admissions and cumulative length of stay (LOS) in the six months post-insertion compared with the six months prior. The median number of admissions per patient also decreased, reflecting a shift toward community-based ascites management.

Table 4 and Figure 2 demonstrate the reduction in hospital utilisation pre- versus post-LTAD insertion.

**Table 4:** Healthcare Utilisation Before and After Long-Term Abdominal Drain (LTAD) Insertion LOS: length of stay. Data represent total admissions and inpatient bed-days for the cohort in the six months before and after LTAD insertion. Relative reductions reflect the proportional change between the two periods.

Measure	6 months before LTAD	6 months after LTAD	Relative Reduction (%)
Total hospital admissions (for cohort)	102 (median 2 per patient)	54 (median 1 per patient)	–47%
Cumulative hospital LOS, days (cohort)	1,150 days	520 days	–55%
Patients with ≥3 admissions in period	12 (27%)	3 (7%)	–75%
Paracentesis procedures in hospital	130	10	–92%

## Discussion

Median survival after LTAD insertion in our study was eight months, with 65% of patients alive at 12 months. These outcomes are somewhat higher than those reported in previous LTAD cohorts by Macken et al. [14,15], possibly reflecting an earlier disease stage in our cohort, as their studies focused primarily on palliative patients. Overall, this underscores that patients with refractory ascites (RA) have limited life expectancy regardless of intervention, reflecting the advanced stage of liver disease in this population. For context, a recent UK analysis reported a one-year transplant-free survival of only ~43% in refractory ascites [16], highlighting the high mortality associated with this condition. Our cohort’s outcomes therefore fall within the expected range, suggesting that while LTADs effectively palliate symptoms, they do not substantially alter disease trajectory - aligning with the understanding that LTADs serve as a bridge to comfort rather than recovery.

SBP occurred in 18% of our patients, and catheter-related infections in 11%. These figures align closely with the pooled infection rate of ~17% reported across 16 studies in a recent systematic review [15]. Prior LTAD studies have typically reported SBP rates between 10-20% and catheter infection rates around 10%, corroborating that infection remains the most frequent complication of LTAD use. Although this risk is an inherent limitation of long-term drainage, strict aseptic technique during insertion and drainage, patient education, and community nursing support can mitigate it. Expert consensus guidelines [12] and national UK surveys [17] consistently identify infection risk as the main deterrent to LTAD adoption. In our practice, most patients received antibiotic prophylaxis, and all infections were successfully treated without any drain-related deaths. This supports the notion that with careful selection and structured follow-up, LTADs can be safely implemented in clinical practice.

We observed a 47% reduction in hospital admissions and a 55% reduction in cumulative hospital LOS after LTAD insertion. These findings mirror previous reports [7,9,14] showing that transitioning ascites management to the outpatient or community setting can markedly reduce hospital utilisation. In our study, LVP procedures in hospital nearly disappeared (falling from 130 to 10 in six months), indicating that LTADs effectively replaced repeated LVPs. This benefits both healthcare systems and patients by reducing hospital dependency and improving comfort. While part of the observed reduction could reflect mortality (as some patients died within the post-LTAD period), the benefit persisted even after excluding those cases, suggesting a genuine effect. Collectively, our data reinforce the potential of LTADs to ease the healthcare burden associated with end-stage liver disease.

From a patient-centred perspective, LTADs provide the opportunity for symptom control in a familiar environment. Although our study did not formally assess quality of life, anecdotal feedback from patients and carers was positive, consistent with findings from the REDUCe (Refining the Role of LTAD in Refractory Ascites) study [8]. However, this autonomy comes with responsibility for self-management and the potential anxiety of dealing with complications. In our centre, close integration between hepatology, palliative care, and community nursing teams was key to ensuring safety and continuity of care. This collaborative model of hospital-based insertion followed by community-led management is increasingly recognised as best practice for palliative LTAD therapy [12].

Limitations and future directions

Our study has limitations. It was retrospective and single-centred with a modest sample size (N = 44), limiting generalisability. The absence of a control group means we cannot directly compare LTADs with ongoing LVP or TIPS. This restricts our ability to infer causality, and the observed reductions in hospital utilisation should therefore be interpreted as associations rather than definitive effects.

The before-and-after design also carries potential for survivor bias and temporal confounding. Because clinical management practices can evolve over time, unmeasured factors such as improved community care or antibiotic use may have contributed to the observed trends.

We mitigated these limitations by using each patient as their own control and confirming that improvements persisted even when early deaths were excluded. Missing or incomplete data were minimal, but variability in documentation across clinicians could introduce minor inconsistencies.

We did not collect formal quality-of-life or symptom-burden metrics, which are crucial in evaluating palliative interventions. Despite these constraints, our findings provide meaningful real-world evidence supporting the feasibility and safety of LTADs in refractory ascites and emphasise the importance of multidisciplinary management.

Future prospective trials are needed to define optimal patient selection, infection-prevention strategies, and cost-effectiveness. Studies such as the ongoing REDUCe-2 trial [6] are expected to clarify LTADs’ impact on quality of life and healthcare resource use. Future work should also investigate safe drainage volumes, as high daily outputs (>1.5 L/day) have been associated with hyponatraemia and renal impairment [18]. Establishing standardised drainage and monitoring protocols will be key to ensuring safety and wider adoption.

Finally, while devices such as the automated low-flow ascites pump (alfapump®; Sequana Medical, Ghent, Belgium) offer an alternative method for continuous ascites removal [5], they remain limited by cost and mechanical complications. Compared with this, LTADs offer a simpler, more affordable option, though both share the challenge of infection control. It remains to be seen whether one approach will predominate or whether both will occupy complementary roles across different patient populations.

## Conclusions

LTADs appear to be a safe and feasible palliative option for patients with RA due to cirrhosis who are unsuitable for TIPS or liver transplantation. In this single-centre service evaluation, LTAD use was associated with meaningful reductions in hospital admissions and length of stay, while complication rates-particularly infections-remained within acceptable and manageable limits. These findings suggest that LTADs can facilitate symptom control and community-based management in carefully selected patients with advanced liver disease. However, these results should be interpreted as observational associations rather than causal effects due to the retrospective design and lack of a control group.

As the burden of cirrhosis continues to rise, LTADs may serve as an important component of palliative care pathways. Careful patient selection and thorough education are essential to ensure safety, given the infection risk. Multidisciplinary, patient-centred management integrating hepatology, palliative care, and community nursing is critical to optimise outcomes with LTAD therapy. Current expert guidance views LTADs as a case-by-case intervention rather than a routine standard of care. Wider adoption will depend on robust prospective controlled research, improved community integration, and standardised protocols to balance the benefits of reduced hospitalisation against infection risk. With thoughtful implementation and ongoing evaluation, LTADs have the potential to improve comfort, dignity, and healthcare efficiency in this high-need population.

Our findings reflect real-world experience within a district general hospital service and provide insight into the practical value of LTADs in palliative ascites management. This service evaluation highlights the feasibility and safety of this approach, offering information that may inform future best practice and ongoing multicentre studies.
